# Time to recovery and its predictors among children aged 6 to 59 months with uncomplicated severe acute malnutrition treated in outpatient therapeutic programs in Adet Woreda, Central Zone of Tigray, Northern Ethiopia: A retrospective cohort study

**DOI:** 10.1371/journal.pone.0359619

**Published:** 2026-09-29

**Authors:** Ashebr Abrha, Solomon Hintsa, Ebud Ayele Dagnazgi, Freweyni Fisshatsion, Goitom Girmay

**Affiliations:** 1 Adet Woreda Health Office, Adet, Central Zone, Tigray, Ethiopia; 2 Department of Epidemiology and Biostatistics, College of Health Sciences, Aksum University, Aksum, Ethiopia; 3 Department of Public Health Nutrition, College of Health Sciences, Aksum University, Aksum, Ethiopia; 4 School of Medicine, College of Health Sciences, Aksum University, Aksum, Ethiopia; 5 Department of Midwifery, College of Health Sciences, Aksum University, Aksum, Ethiopia; University of Oulu, FINLAND

## Abstract

**Background:**

Severe acute malnutrition remains a major public health problem and a leading cause of under-five morbidity and mortality in low and middle income countries. Although outpatient therapeutic feeding programs are widely used to treat uncomplicated cases, recovery time often remains below SPHERE standards. Evidence on time to recovery and its predictors is limited in Tigray, particularly in post-conflict settings where health services have been disrupted.

**Objective:**

To determine the time to recovery and identify its predictors among children aged 6–59 months with uncomplicated severe acute malnutrition treated in outpatient therapeutic programs in Adet woreda.

**Methods:**

A retrospective cohort study was conducted among children aged 6–59 months with uncomplicated severe acute malnutrition treated in outpatient therapeutic programs from March 2023 to February 2024. A total of 479 children were selected by systematic random sampling. Bivariable Cox proportional hazards regression was performed to identify candidate variables, and variables with a p ≤ 0.25 were entered into the multivariable model. Statistical significance was declared at p < 0.05.

**Results:**

The overall recovery rate was 90.8%, with a median recovery time of 49 days. Independent predictors of recovery time were sex (AHR = 1.27, 95% CI:1.04, 1.54), travel time to health facility (AHR = 0.57, 95% CI: 0.44,0.74), residence (AHR = 0.52, 95% CI: 0.42, 0.64), vomiting at admission (AHR = 2.48, 95% CI: 1.27, 4.83), pneumonia at admission (AHR = 3.06, 95% CI: 1.83, 5.11), admission status (AHR = 0.52, 95% CI: 0.39, 0.71), and admission diagnosis (AHR = 0.54,95% CI:0.36,0.81).

**Conclusions:**

The recovery rate of children with severe acute malnutrition treated in outpatient therapeutic feeding programs exceed the minimum standard. Early identification and management of medical illnesses, improved access to health facilities, and strengthened collaboration between governmental and nongovernmental organizations are essential reduce recovery time among children receiving outpatient therapeutic programs services.

## Introduction

Severe acute malnutrition (SAM) is defined as presence of bilateral pitting edema, a mid upper arm circumference of less than 115 mm, or a weight for height below Z score below −3 according to the World Health Organization (WHO) child growth standards. These criteria are widely used for the identification and management of SAM in both community and clinical settings. SAM remains a major global public health problem, particularly in low and middle income countries, and is strongly associated with increased morbidity, mortality, impaired growth, and poor cognitive development among children under five years of age [1,2].

Globally, an estimated 13 million children are affected by SAM every year [3]. The burden is high in Africa, where approximately 12.7 million children are affected by moderate and severe wasting, including about 3.5 million with severe wasting. SAM remains a major contributor to under-five mortality, accounting for 11% of deaths among affected children [4]. In sub-Saharan Africa, about 11.8 million children experience moderate and severe wasting, including 3 million with SAM, and the recovery rate among children treated for SAM is about 71.2%, which is below the minimum SPHERE standard [5,6].

In Ethiopia, the prevalence of SAM among children under five years of age is estimated to be 8% [7]. The mortality rate among children with SAM admitted to outpatient therapeutic feeding programs (OTP) is around 2% [8]. In addition, the median recovery time is often longer than the recommended SPHERE standards [9]. In Tigray, the burden of malnutrition remains high, with reported prevalence of severe, moderate, and overall global acute malnutrition 5.1%, 21.8%, and 26.9%, respectively [10]. The recovery rate among children treated for SAM in sub Saharan Africa, including Ethiopia, is 71.2%, which is below the minimum acceptable level defined by SPHERE standards [6]. Furthermore, in Ethiopia, the overall mean length of stay, defaulter rate, and mean weight gain among children enrolled in OTP programs do not consistently meet the SPHERE standards [8].

SAM is classified as either complicated or uncomplicated. Complicated SAM refers to children with clinical conditions such as poor appetite, intractable vomiting, convulsions, lethargy, altered consciousness, high fever, lower respiratory tract infection, dehydration, persistent diarrhea, severe anemia, hypoglycemia, and hypothermia [11]. In contrast, uncomplicated SAM refers to children who are clinically stable, have no signs of severe infection or other indications for hospital admission, and demonstrate adequate appetite during the appetite test [12].

Several factors have been reported to predict the recovery time among children with SAM in outpatient settings. These include comorbidities such as diarrhea, anemia, and pneumonia, as well as the type of malnutrition, provision of antibiotics, immunization status, and deworming practices [6,13,14].

Despite the high burden of SAM, evidence on the recovery time and its predictors among children with SAM treated in OTP sites remains limited in the Central Zone of Tigray, particularly in Adet Woreda. Moreover, there is a lack of studies conducted before and after the war that assess treatment outcomes and recovery patterns in Adet Woreda despite the substantial disruption of health services. Therefore, this study aimed to assess the recovery time and identify its predictors among children aged 6–59 months with uncomplicated SAM treated in OTPs in Adet Woreda.

## Materials and methods

### Study setting and period

This study was conducted in Adet woreda, located in the Central Zone of Tigray, northern Ethiopia. The woreda was among the areas affected by the armed conflict in Tigray (2020–2022), which severely disrupted health service delivery, damaged health infrastructure, interrupted routine maternal and child health services, and limited access to nutrition programs, including OTPs. Following the cessation of active conflict, health services were gradually restored with support from governmental and humanitarian organizations. At the time of the study, OTP services for children with uncomplicated SAM were provided through one primary hospital, four health Centers (HC), 17 health posts (HP), and one private clinic. The primary hospital, all health centers and most health posts provide OTP services for children with uncomplicated SAM. The study was conducted from 01/03/2023–29/02/ 2024.

### Study design

A health facility based retrospective cohort study was conducted.

### Source population

All children aged 6–59 months who were diagnosed with uncomplicated SAM and treated at OTP sites in health facilities of Adet woreda.

### Study population

All children aged 6–59 months who were diagnosed with uncomplicated SAM and treated at OTP sites in the selected health facilities of Adet woreda.

### Eligibility criteria

#### Inclusion criteria.

All medical records of children aged 6–59 months who were diagnosed with uncomplicated SAM and admitted to OTP sites during the study period were included.

#### Exclusion criteria.

Medical records with incomplete information on key variables and records of children with SAM who were transferred from other facilities to the selected OTP sites without complete referral documentation were excluded.

### Sample size determination

The sample size was calculated using STATA version 14 based on survival analysis assumptions for the Cox proportional hazards model. The calculation was based on a 5% level of significance, and 80% statistical power. The probability of the event was assumed to be 0.54, based on a previous similar study conducted in Southern Ethiopia, and the assumed hazard ratio of 1.55 [9]. The required number of events was calculated using the formula;


$$\begin{array}{c} \text{E}=\;{\left({\text{Z}}_{a}+\beta \right)}^{\text{2}}/{\text{Ln }(\text{HR})}^{\text{2}}\text{ q0x }{\text{p}}_{\text{1}} \end{array}$$

Where; N = Total required Sample size

E = Number of events (recovery)

P (E) = Overall probability of observing events in the study population

α = Type I error

β= Type II error

p_1_ = Probability in the exposed group

q_0_ = Probability in the unexposed group

HR = Hazard risk

The total sample size was then calculated using;


$$\begin{array}{c} N=E/P(E) \end{array}$$

The initial calculated sample size was 303. After adding 5% to compensate incomplete records and applying a design effect of 1.5, the final sample size was 479 children.

### Sampling technique and procedure

A multistage sampling technique was employed to select study participants. Initially, all 17 OTP sites in Adet woreda were identified. These include 12 health posts, four health centers, and one primary hospital. From these facilities, eight OTP sites were selected using simple random sampling.

Following the selection of the study sites, the total sample size was proportionally allocated to each selected facility based on the number of eligible children aged 6–59 months with uncomplicated SAM registered at each selected site. The number of eligible children was obtained from the OTP registration logbooks of each health facility.

Accordingly, the allocated sample sizes were as follows: Debre Genet HC (n = 60), Jira HC (n = 79), Edaga Berhe HC (n = 118), Adi Selam HP (n = 69), May Danya HP (n = 33), Adeke Bekli HC (n = 70), Gito HP (n = 12), and Sabe Ruba Adet HP (n = 38).

Finally, systematic random sampling was applied at each selected facility. Eligible children were selected from the registration logbooks using sampling interval (K) of two, which was determined by dividing the total number of eligible children by the allocated sample size. The first record was randomly selected using lottery method, and every second record was included thereafter until the required sample size for each facility. This resulted in a total sample size of 479 children and ensured proportional representation across the selected the selected facilities.

### Operational definitions

**Censored**: A child not recovered from SAM. This includes transfer out, medical transfer, defaulter, death and non-responder.

**Died**: A patient died while receiving treatment in the OTP Program [11].

**Defaulter**: A Patient that was absent for two consecutive weightings (14 days in outpatient), confirmed by a home visit for the outpatient component of the program [11].

**Non-responder**: do not reach the SAM discharge criteria after 16 weeks/4/months of treatment of treatment [11].

**Medical transfer**: A Patient that was referred to a health facility/ hospital for medical reasons. And this health facility will not continue the nutritional treatment or transfer the patient back to the program [11].

**Recovery rate**: Number of patients discharged for recovery / Total No of exits [11].

**New**: New case who meets the admission criteria for OTP [11].

**Readmission**: Readmitted to treatment in OTP due to relapse or returned defaulter who meets the admission criteria [11].

**Recovery time**: Number of days it takes from admission until a child is recovered from SAM. It is four weeks according acceptable sphere standards [15].

**Incomplete data**: Medical records missing key variables like admission date, discharge date, or treatment outcome.

### Data collection tool and procedure

Data were extracted from the OTP registration logbooks and patient records using a structured and pretested data extraction checklist adapted from a previous similar studies [16,17]. The checklist included socio-demographic, clinical, and treatment related variables. Data extraction was performed by three trained clinical nurses under the supervision of one Bachelor of Science nurse. The principal investigator and the supervisor conducted regular monitoring and supervision throughout the data collection period. Completed data extraction forms were reviewed daily for completeness, consistency, and accuracy. Any missing or inconsistent information was corrected by revisiting the medical records.

### Data quality control

Data quality was maintained throughout all stages of the study. The data extraction checklist was developed after reviewing relevant literatures and standardized guidelines. The tool was initially prepared in English and translated into Tigrigna to ensure consistency. However, the English version was used for data extraction because OTP records were documented in English. The consistency and clarity of the tool were reviewed by a language expert. Experienced data collectors and supervisors was recruited and training was provided for two days on the objectives of the study, data collection procedures, and confidentiality of information. Pretest was conducted on 5% of the sample size in other OTP site in the woreda that was not included in the actual study.

The collected data was checked for completeness by supervisors on a daily basis. Consequently, any problem encounter was discussed among the team and solved immediately.

### Data processing and analysis

The collected data were checked for completeness, coded, and entered into EpiData version 4.4.2 and then exported to STATA version 14 for analysis. Exploratory data analysis was conducted to assess missing values, influential outliers, Multicollinearity, normality, and proportional hazards assumption. Descriptive statistics such as frequencies, percentages, tables, and graphs were used to summarize categorical variables, while mean and median were used for continuous variables. The Kaplan-Meir survival analysis method was used to estimate the median recovery time, and differences between groups were assessed using the log rank test. Both bivariable and multivariable Cox proportional hazards regression models used to identify predictors of recovery time. Variables with a P ≤ 0.25 bivariable Cox proportional hazards regression analysis were entered into the multivariable Cox proportional hazards regression model. Statistical significance was declared at P < 0.05, with Adjusted Hazard Ratios (AHR) and 95% CI used to measure strength of association. The proportional hazards assumption was evaluated using assessed scientifically by Schoenfeld residual’s test and graphically using log minus log survival curves. The Schoenfeld residual’s test result of 0.057 indicated that the proportional hazards assumption was satisfied.

### Ethical considerations

Ethical clearance was obtained from the Ethical Review Committee (IRB/063/2024) of Aksum University, College of Health Sciences. An official permission letter was provided by Aksum University College of Health Sciences and submitted to the Adet Woreda Health Office. Permission to conduct the study was obtained from the woreda health office and selected health facilities. Since the study was based on retrospective review of medical records, informed consent was not required from mothers or care givers. Confidentiality of the data was maintained throughout the study by using anonymous data collection tools.

## Results

### Socio-demographic characteristics and treatment outcomes

A total of 479 children aged 6–59 months with uncomplicated SAM were included in the study. The overall recovery rate was 90.8% (435/479), while 9.2% (44/479) were censored. The median time to recovery was 49 days with an interquartile range of 21 days. More than half (51.8%) of the participants were female and while 48.2% were male. The majority (86.4%) of children were younger than 24 months. Most participants (58.2%) were from rural areas and 78.5% traveled less than one hour to reach the health facility. The recovery rate for females and males were 91% and 90.4% respectively. The recovery rate for children less than 24 months and greater 24 months were 90.3% and 93.8% respectively (Table 1).

**Table 1:** Socio-demographic and health service related characteristics of children aged 6 to 59 months with SAM enrolled in the OTP in Adet Woreda, Central Zone of Tigray, Ethiopia, 2024.

Variables	Categories	Event to recovery		Total (%)
		Recovered	Censored	
Time of travel	< 1 hour	346(92.0%)	30(8.0%)	376(78.5%)
	≥1 hour	89(86.4%)	14(13.6%)	103(21.5%)
Residence	Urban	189(94.5%)	11(5.5%)	200(41.7%)
	Rural	246(88.2%)	33(11.8%)	279(58.3%)
Age	<24 months	374(90.3%)	40(9.6%)	414(86.4%)
	≥24 months	61(93.8%)	4(6.2%)	65(13.5%)
Sex	Male	209(90.4%)	22(9.6%)	231(48.2%)
	Female	226(91.0%)	22(9.0%)	248(51.8%)
Referred by	Community health worker	365(92.1%)	31(7.9%)	396(82.6%)
	Others	70(84.3%)	13(15.7%)	83(17.4%)

### Comparison of survival functions for children with SAM treated at OTP (The Kaplan-Meier survival analysis)

Children who traveled less than one hour to reach to the OTP sites had shorter median recovery time compared to those who traveled one hour or more (49 days versus 56 days). The difference in recovery time between those who traveled less than one hour and who traveled one hour or more was statistically significant (log-rank = 34.6, p < 0.001) (Fig 1 and Table 2).

**Table 2:** Kaplan-Meir estimates of median recovery time by selected covariate and log rank test among children aged 6-59 months with SAM treated in OTP in Adet Woreda Central Zone of Tigray, Ethiopia, March 2023 to February 2024.

Variables	Categories	Recovered	Censored	Median recovery time	Log rank	P- value
Time of travel	<1hr	346	30	49	34.60	< 0.001
	≥1hr	89	14	56		
Residence	Urban	189	11	40	56.90	< 0.001
	Rural	246	33	51		
Vomiting	Yes	9	6	60	9.25	0.002
	No	426	56	49		
Pneumonia	Yes	17	7	76	23.70	< 0.001
	No	418	37	48		
Admission status	New	413	27	48	24.62	< 0.001
	Readmission	9	11	76		
Sex	Male	209	22	50	4.65	0.030
	Female	226	22	46		
Diagnosis at admission	Marasmus	416	33	49	4.70	0.040
	Kwashiorkor	20	10	55		

**Fig 1:**
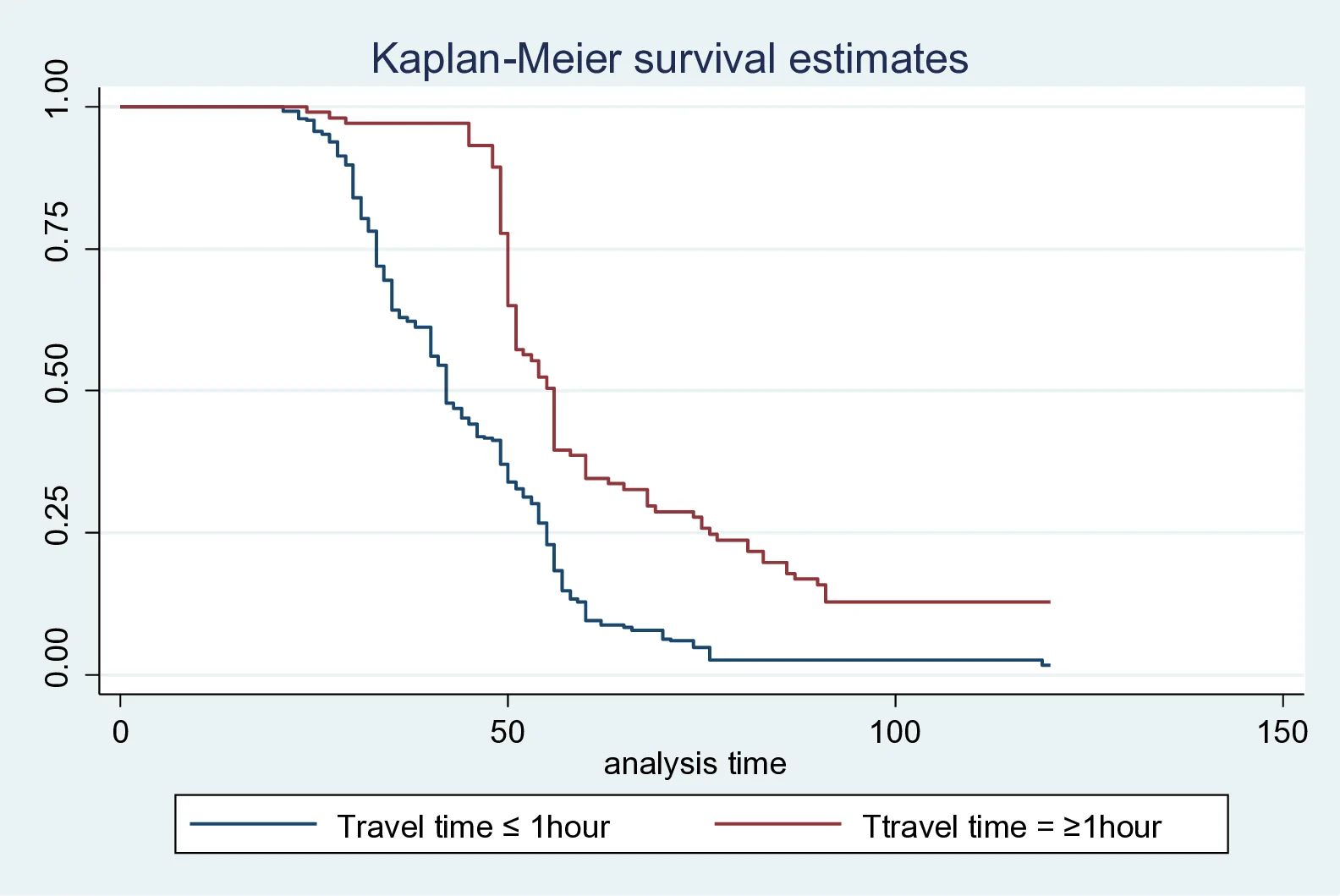
Kaplan-Meir curve showing time to recovery among children aged 6-59 months with SAM childrens by travel time to OTP sites in Adet Woreda, Central Zone of Tigray, Ethiopia, March 2023 to February 2024.

Children residing in urban areas had a shorter median recovery time compared with those from rural areas (40 day versus 51 days respectively). The difference was statistically significant (log-rank = 56.9, p = 0.014) (Fig 2 and Table 2).

**Fig 2:**
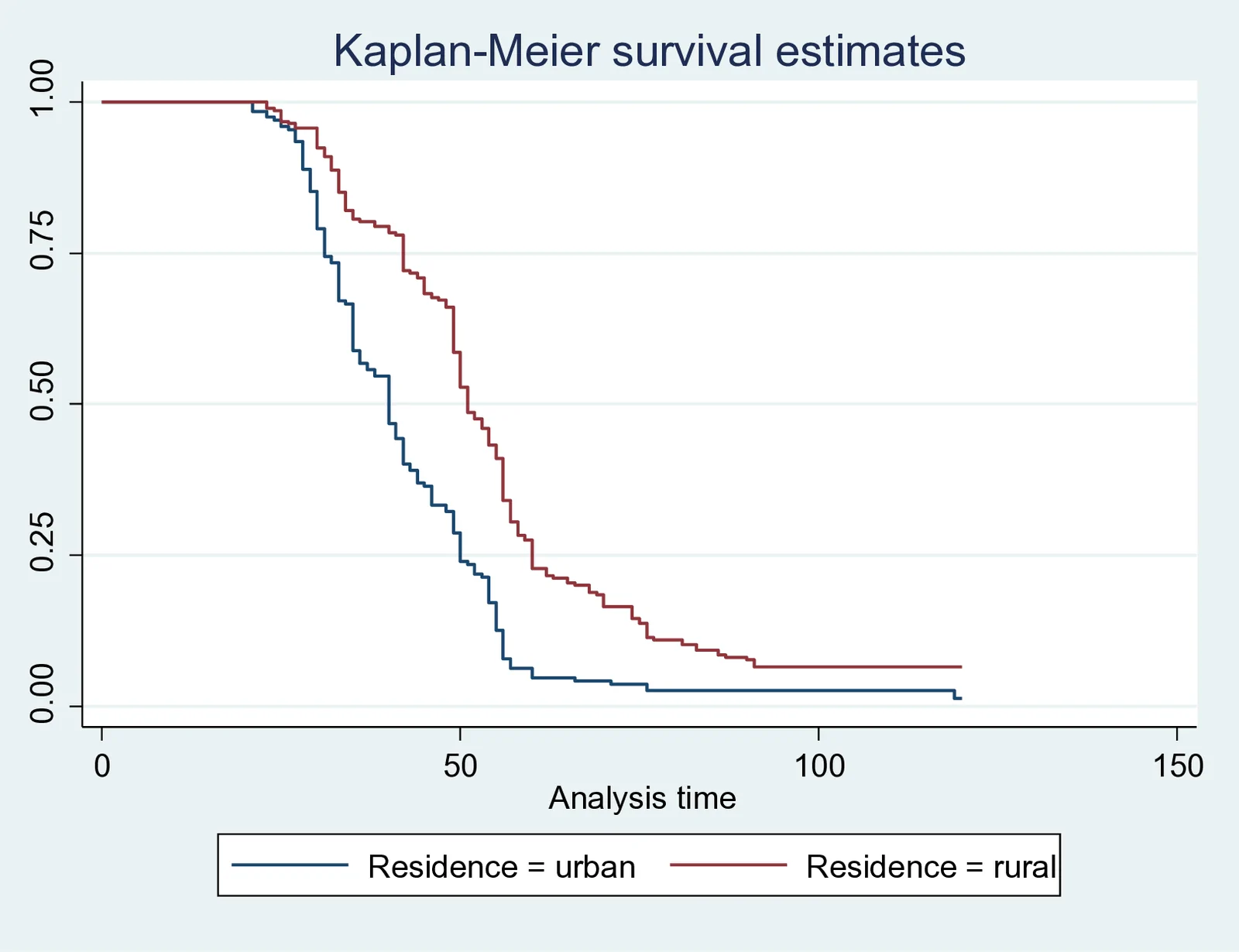
Kaplan-Meir curve showing time to recovery among children aged 6-59 months with SAM childrens by place of residence in OTP sites in Adet Woreda, Central Zone of Tigray, Ethiopia, March 2023 to February 2024.

The Kaplan Meier survival analysis also showed that children without vomiting at admission had a shorter median recovery time compared with presented with vomiting at admission (49 days versus 60days recovery). This difference was statistically significant (log rank = 9.25, p = 0.0023) (Fig 3 and Table 2).

**Fig 3:**
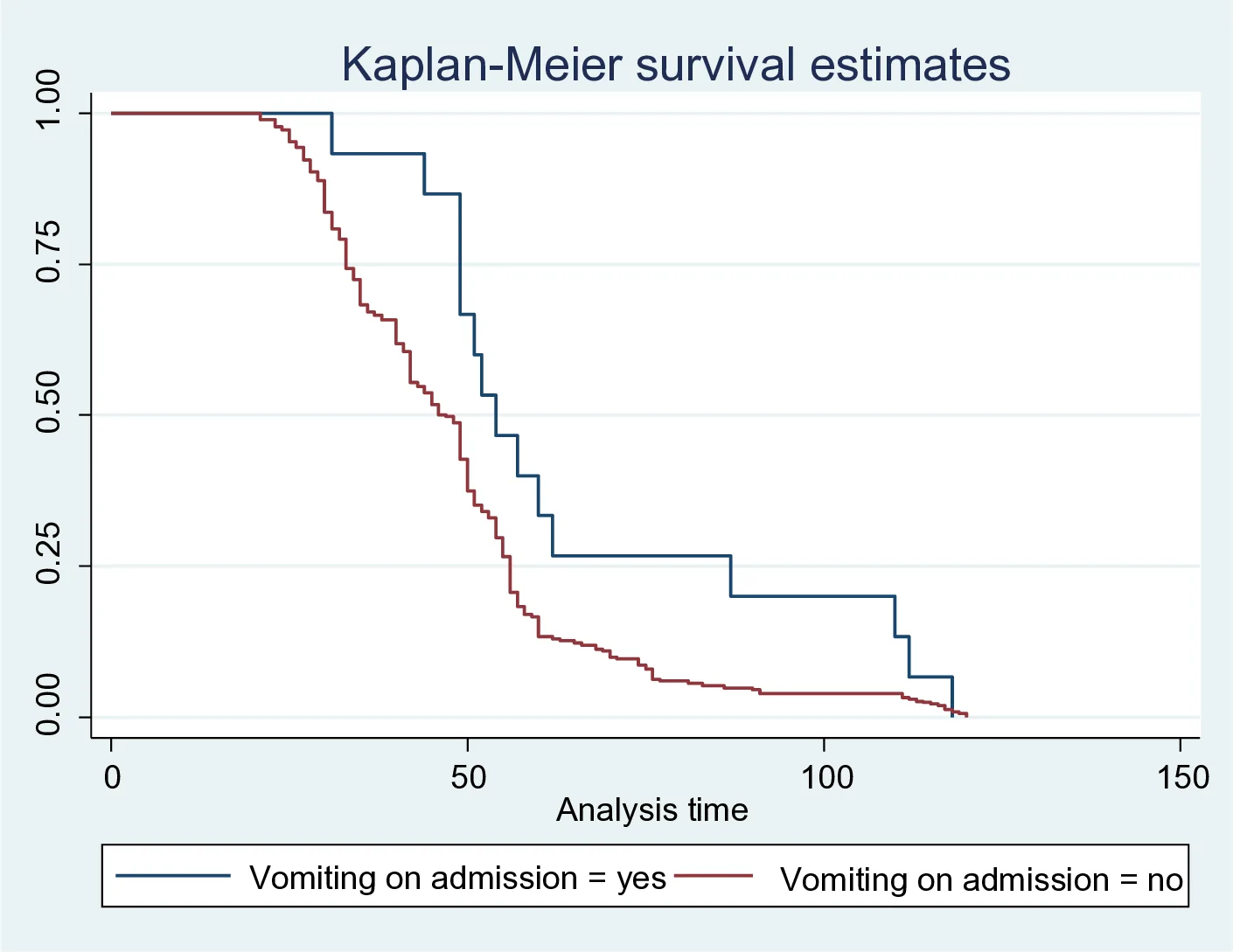
Kaplan-Meir curve showing time to recovery among children aged 6-59 months with SAM childrens by vomiting at admission in OTP sites in Adet Woreda, Central Zone of Tigray, Ethiopia, March 2023 to February 2024.

Regarding admission status, newly admitted children had shorter median recovery time compared with readmitted children (48 days versus 76 days). The difference between newly admitted and readmitted was statistically significant (log-rank = 24.62, p < 0.001) (Table 2).

Children without pneumonia recovered faster than children with pneumonia with median recovery time of 48 days versus 76 days respectively. The difference between the groups was statistically significant (log-rank = 23.70, p < 0.001) (Fig 4 and Table 2).

**Fig 4:**
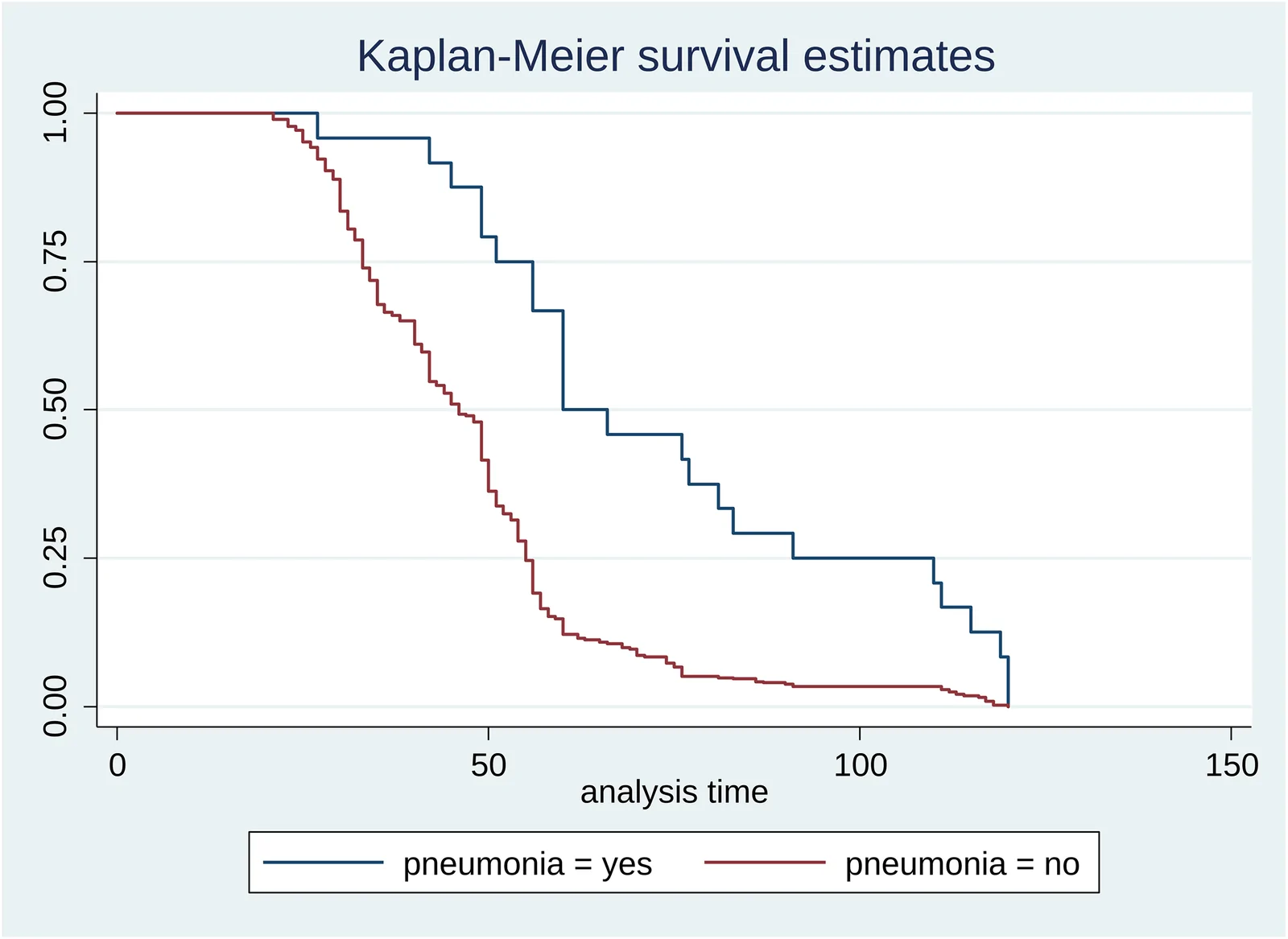
Kaplan-Meir curve showing time to recovery among children aged 6-59 months with SAM childrens by pneumonia in OTP sites in Adet Woreda, Central Zone of Tigray, Ethiopia, March 2023 to February 2024.

Similarly, female children had shorter median recovery time compared with male children (46 days versus 50 days) and the difference was statistically significant (log-rank = 4.65, p < 0.030) (Fig 5 and Table 2).

**Fig 5:**
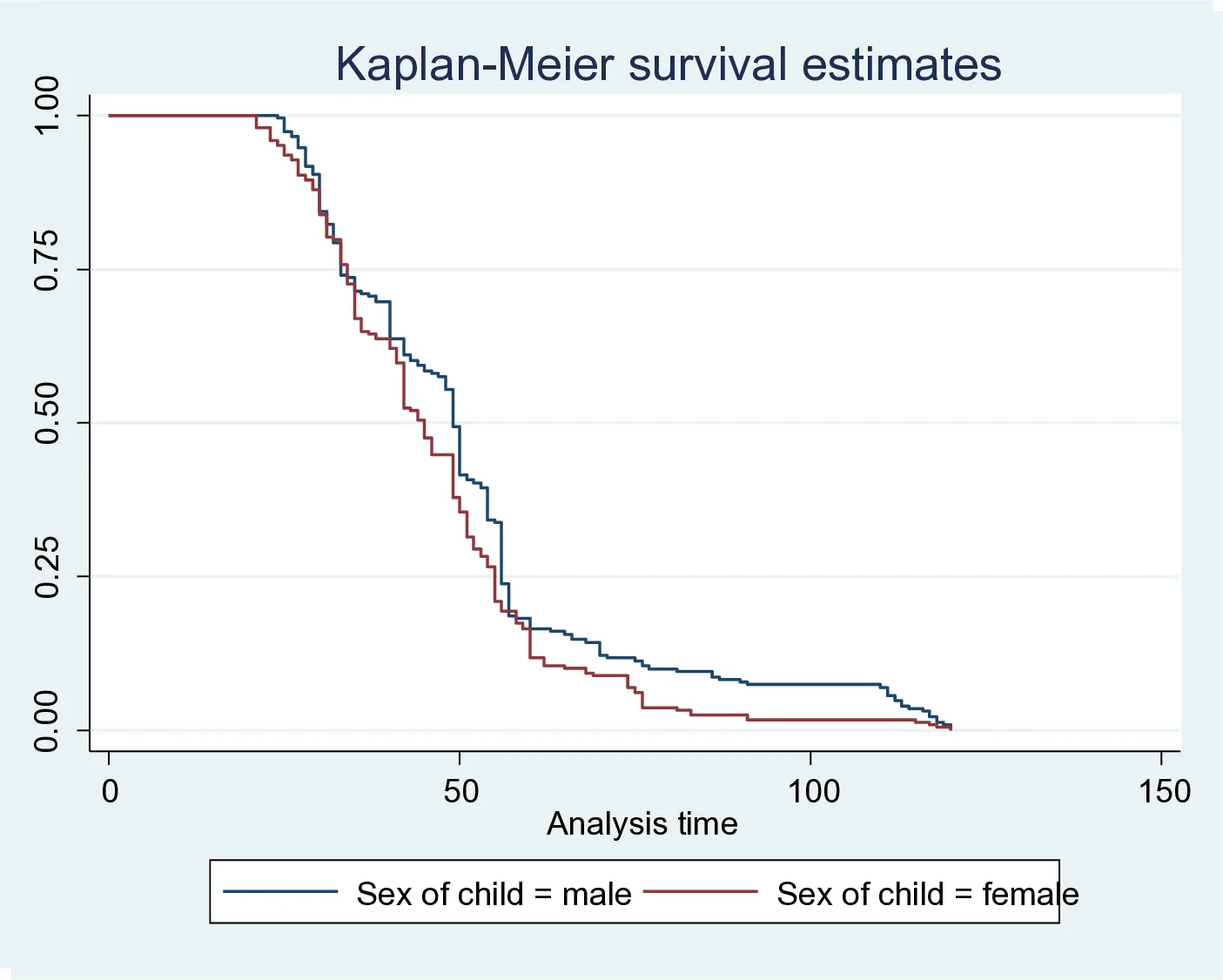
Kaplan-Meir curve showing time to recovery among children aged 6-59 months with SAM childrens by sex in OTP sites in Adet Woreda, Central Zone of Tigray, Ethiopia, March 2023 to February 2024.

In addition, children diagnosed with marasmus had a shorter median recovery time compared with those with kwashiorkor (49 days versus 55 days respectively and the difference was statistically significant (log-rank = 4.70, p < 0.040) (Table 2).

### Factors associated with the recovery time among children treated in OTP

Bivariable Cox proportional hazards regression analysis was performed to identify candidate variables for multivariable analysis. Variables with p ≤ 0.25 were considered for the final multivariable Cox proportional hazards model. These include residence, sex, travel time to health facility, admission status, vomiting at admission, malaria, pneumonia, measles immunization, deworming status, admission criteria, diagnosis at admission. The proportional hazards assumption was assessed using the Schoenfeld residuals test, and the model satisfied the assumption (global test p = 0.057). In multivariable analysis, sex (AHR = 1.27, 95% CI: 1.04–1.54), travel time (AHR = 0.57, 95% CI: 0.44,0.74), residence (AHR = 0.53, 95% CI: 0.42–0.64), vomiting at admission (AHR = 2.48, 95% CI: 1.27–4.81), pneumonia at admission (AHR = 3.06, 95% CI: 1.83–5.11), admission status (AHR = 0.52, 95% CI: 0.39–0.71), and diagnosis at admission (AHR = 0.54, 95% CI: 0.36–0.81) were significantly associated with recovery time (Table 3).

**Table 3:** Proportional hazards cox model multiple variable analyses of determinants of survival /time of recovery of SAM children in OTP in Adet woreda, Central Zone of Tigray, Ethiopia from March 2023 to February 2024.

Variables	Categories	Recovered	Censored	CHR (95% CI)	P- Value	AHR(95% CI)	P – Value
Sex	Male	209	22	1	0.038	1	0.016
	Female	226	22	1.22(1.01,1.47)		1.27(1.04,1.54)	
Time of travel	< 1 hour	346	30	1	0.000	1	0.000
	≥ 1 hour	89	14	0.44(0.34,0.55)		0.57(0.44,0.74)	
Residence	Urban	189	11	1	0.000	1	0.000
	Rural	246	33	0.43(0.40,0.59)		0.52(0.42,0.64)	
Vomiting	Yes	9	6	1	0.005	1	0.007
	No	426	56	2.58(1.33,5.01)		2.48(1.27,4.83)	
Pneumonia	Yes	17	7	1	0.000	1	0.000
	No	418	37	3.05(1.86,4.99)		3.06(1.83,5.11)	
Admission status	New	413	27	1	0.000	1	0.000
	Readmission	9	11	0.51(0.38,0.68)		0.52(0.39,0.71)	
Admission diagnosis	Marasmus	416	33	1		1	0.003
	Kwashiorkor	20	10	0.66	0.040	0.54(0.36,0.81)	

AHR, Adjusted Hazard Ratio; CHR, Crude Hazard Ratio; CI, Confidence Interval.

## Discussion

In this study, 435(90.8%) children with SAM recovered following treatment in the OTP. This recovery rate exceeded the minimum SPHERE standard of 75% [15]. The recovery rate was also higher than the rates reported in previous studies conducted in the Tigray region (76.8%), Afar regional state (67.7%), and Kamba, Southern Ethiopia (61.78%) [18–20]. Despite the disruption of health services caused by the recent conflict in the region, the high recovery rate observed in this study may reflect the ongoing restoration of the health services, increased governmental and nongovernmental support, and the commitment of healthcare providers to delivering quality outpatient therapeutic care.

In the present study, the proportion of non-responders, defaulters, and deaths were 4.2%, 1.5%, and 0.2% respectively. These findings were within the acceptable SPHERE standards [15]. Furthermore, the defaulter, non-responder, and mortality rates observed in this study were lower than those reported in studies conducted in Afar Regional state (6.3%, 4.9%, and 2.8% respectively) [19], and the Tigray region (13.85% defaulter rate, and 3.02% mortality rate) [18].

However, the median recovery time in this study was 49 days, which exceeded the SPHERE recommended recovery time of less than 28 days [15]. This recovery time was longer than that reported in the Afar regional state (44.15 days) but comparable to the finding from Shebedino Woreda in Sidama region (49 days) [19,21]. The prolonged recovery time observed in this study may be attributable to suboptimal consumption of ready to use therapeutic food, possibly due to sharing within households or diversion for non-therapeutic purpose. Another possible reason for the prolonged recovery time in the children may be due to the presence of comorbidities and delayed heath seeking behavior.

In this study, children who traveled for more than one hour to reach the treatment center had a significantly lower recovery rate compared to those who traveled for one hour or less. This finding is consistent with a study conducted in north Shewa Zone, Oromia region [22]. A possible explanation is that longer travel time may delay access to timely care, reduced adherence to scheduled follow up visits, and reflect underlying socio-economic and geographic barriers.

Children without vomiting at admission had a significantly higher recovery rate than those who presented with vomiting. This finding is consistent with the study conducted in North Gondar, Ethiopia, which reported that vomiting at admission was associated with prolonged recovery time [23]. A possible explanation is that vomiting may reduce therapeutic food intake and impair nutrient absorption, thereby delaying nutritional rehabilitation.

Newly admitted children had a significantly higher recovery rate compared to readmitted children. This finding is consistent with the studies conducted in Arsi Negele, Oromia region and Bench Shoko Zone, Southwest Ethiopia [9,24]. The difference may be explained by better treatment responsiveness and fewer underlying complications among newly admitted children, whereas readmitted children may have persistent risk factors, poor treatment adherence, or recurrent illness.

Children without pneumonia at admission had a significantly higher recovery rate than those with pneumonia. A similar finding was reported in a study conducted in Amhara region, which found that pneumonia was associated with delayed recovery among children with SAM [25]. The presence of pneumonia may increase metabolic demand, reduce appetite, and impair the body’s response to nutritional therapy, thereby prolonging recovery time.

Admission diagnosis was also found to be a significant predictor of recovery time. Children admitted with kwashiorkor had a lower recovery rate than those diagnosed with marasmus. This finding is supported by a study conducted in WagHimra Zone, Northeast Ethiopia [26]. Children with kwashiorkor are more likely to have complications such as infections, electrolyte imbalance, and poor appetite, all of which may delay nutritional recovery compared with children with marasmus [27].

## Conclusions

The recovery rate among children with SAM treated in OTPs exceeded the minimum international SPHERE standard. However, the median recovery time was longer than the recommended SPHERE standard. Sex, travel time to the health facility, place of residence, vomiting, pneumonia, admission status, and admission diagnosis were significant predictors of recovery time.

Strengthening the early identification and management of medical illnesses is essential to reduce the duration of treatment among children enrolled in OTPs. Improving access to health facilities, particularly for children who travel long distances, may also improve treatment outcomes. In addition, collaborative efforts between governmental and nongovernmental organizations are needed to strengthen the quality of OTPs, improve treatment effectiveness, and shorten recovery time among children with SAM.

## Limitation of the study

Since this study used a retrospective facility based study design, some important variables were not included in the medical records. These variables include, adherence to ready to use therapeutic food consumption and key paternal and child related factors such as paternal and maternal educational status, breastfeeding practices, and maternal nutritional status were not included.
